# Acknowledgment to Reviewers of *Journal of Cardiovascular Development and Disease* in 2021

**DOI:** 10.3390/jcdd9020045

**Published:** 2022-01-28

**Authors:** Journal of Cardiovascular Development and Disease Editorial Office

**Affiliations:** MDPI AG, St. Alban-Anlage 66, 4052 Basel, Switzerland

Rigorous peer-reviews are the basis of high-quality academic publishing. Thanks to the great efforts of our reviewers, *Journal of Cardiovascular Development and Disease* was able to maintain its standards for the high quality of its published papers. Thanks to the contribution of our reviewers, in 2021, the median time to first decision was 19 days, and the median time to publication was 39.5 days. The editors would like to extend their gratitude and recognition to the following reviewers for their precious time and dedication, regardless of whether the papers they reviewed were finally published:
Afanasieva, OlgaLorenzo González, ÓscarAgrifoglio, MarcoLozano-Velasco, EstefaniaAl-Adawi, SamirLucchese, GianlucaAlexy, TamasLucero, DiegoAmarelli, CristianoLugnier, ClaireAmiya, EisukeLundegaard, Pia RengtvedAndreassi, MariaMa, HongAngeli, FabioMa, HuiAngelini, PaoloMacedo, GuilhermeAntonakopoulos, NikolaosMahmoud, AhmedArnaud, PaulineMahtab, Edris A. F.Azhar, MohamadMair, HelmutBajraktari, GaniMałek, ŁukaszBalla, CristinaMaloberti, AlessandroBamforth, Simon D.Maltsev, VictorBaranyai, TamásMancarella, SalvatoreBarge-Schaapveld, Daniela Q. C. M.Manesis, Emanuel K.Barison, AndreaManetti, MirkoBarletta, GiuseppeMantovani, FrancescaBarragán, RocioMarín-Juez, RubénBayes De Luna, AntonioMarino, FabiolaBeis, DimitrisMariotti, AlessandroBelo, José AntónioMarkwald, RogerBelo, LuisMartini, BortoloBergau, LeonardMasarone, DanieleBianco, FrancescoMassaro, MarikaBidaux, GabrielMattina, AlessandroBody, Simon ChristopherMatusik, PawełBoriani, GiuseppeMaurea, NicolaBorja, Mark S.Mauro, PepiBorovac, Josip A.Mehboob, HassanBrandt, Mathias C.Merkely, BélaBrugada, PedroMetzinger, LaurentBruno, PiergiorgioMeune, ChristopheBusjahn, AndreasMiano, JosephButcher, JonathanMitsui, ToshiyukiCai, QiangjunMiyagawa-Tomita, SachikoCalabrò, PaoloMöller, SörenCalvo-Iglesias, Francisco EugenioMommersteeg, MathildaCaminiti, GiuseppeMontecucco, FabrizioCampione, MarinaMoreno-Macías, HortensiaCapoccia, MassimoMori, ShumpeiCarles Escola-Gil, JoanMoritz, AntonCarulli, LuciaMurdaca, GiuseppeCarvalho, Marta S.Nasso, GiuseppeCastellani, ChiaraNatsis, KonstantinosCazzola, RobertaNeef, KlausChakrabarti, MrinmayNelson, Rachael K.Chang, Chuang-RungNg, G. AndréChen, Chun-JungNguyen, Hoang H.Chen, ShaojieNicolosi, Gian LuigiChen, WeichengNistri, StefanoChen, WeijieNorris, RussellChilton, RobertNurzynska, DariaChiu, NicholasObal, DetlefChmielewski, PrzemyslawObama, TakashiCho, KazutoshiObrist, DominikChokshi, AalapOh, Yong-SeogChristian, MosimannOhte, NobuyukiCimmino, GiovanniOkovityi, Sergey V.Claesen, JürgenOláh, AttilaClark, StephenOliver, EduardoClerico, AldoOmiya, KazutoCollins, Michelle M.Ortega, Miguel A.Conlon, FrankOrtolani, FulviaCosta, MarcoOsuna, EduardoCoufal, NicoleOury, CécileD’Agostino, DonatoParaskevaidis, IoannisD’Angelo, EmanuelaParikh, Priyanka A.Danilowicz-Szymanowicz, LudmilaPark, Hun-JunDas, Bibhuti B.Pasta, SalvatoreDe Geest, BartPastori, DanieleDe Oliveira, Camila MacielPatarrão, RitaDe Santo, Luca SalvatorePatschan, DanielDel Monte Nieto, GonzaloPavasini, RitaDettlaff-Pokora, AgnieszkaPedretti, Roberto Franco EnricoDi Mauro, MichelePellet-Many, CarolineDi Mise, AnnaritaPenson, PeterDi Nora, ConcettaPepi, MauroDiab, MahmoudPerek, BartłomiejDiMizio, GiulioPerrone, Marco AlfonsoDonatelli, FrancescoPetit, Patrice X.Donoiu, IonutPfuhl, GeritDu, XiaoyanPiccoli, MarcoDudzinski, David M.Pitsis, Antonios A.Duran-Ruiz, Mª CarmenPitta, Sridevi R.El-Khazragy, NashwaPlant, LeighEscolà-Gil, Joan CarlesPłatek, Anna E.Etchevers, Heather C.Pöling, JochenFaletra, FrancescoPoniedziałek-Czajkowska, ElżbietaFalsini, GiovanniPorta, AlbertoFang, YuehuaPorter, GeorgeFarre, NuriaPoulsen, Steen HvitfeldtFeier, HoreaPřeček, JanFernández, BorjaProtonotarios, AlexandrosFilipovic, NenadPrsa, MilanFishman, GlennPtaszynska-Kopczynska, KatarzynaFormica, FrancescoPuddu, Paolo EmilioFragakis, NikolaosPulli, RaffaeleFrancisco, Julio CesarPuślecki, MateuszFrancois, MathiasRader, FlorianFriedrich Boettcher, MichaelRahman, AzizFrigy, AttilaRajpurohit, SurendraFukuda, HirotsuguRakowski, TomaszFusini, LauraRammos, ChristosGaba, PrakritiRao, Carmelo MassimilianoGalatou, EleftheriaRav Acha, MosheGallagher, Mark M.Reffo, ElenaGalli, ElenaRegazzoli, DamianoGambaryan, StepanRichards, Dylan JackGao, GeRichardt, DoreenGarrity, DeborahRigby, MichaelGasperetti, AlessioRiley, PaulGatzoulis, Konstantinos A.Rivinius, RasmusGeorge, RajaniRoberto, MaurizioGeorges, AdrienRoberts, Drucilla J.Giannouli, StavroulaRoll, PatriceGilsanz, FernandoRoohafza, HamidrezaGirao, HenriqueRubino, Antonino S.Gonzalez Rosa, Juan ManuelRugonyi, SandraGonzalez, Daniel R.Rybtsov, StanislavGordon, KarenSaeed, SahraiGragnano, FeliceSaid, SamehGrajek, StefanSaito, AyaGrapow, MartinSak, Jarosław J.Greco, ErnestoSakurai, Toshihiro SakuraiGrinshtein, Yuriy I.Salto-Gonzalez, RafaelGrossfeld, PaulSamidurai, ArunGuillen-Grima, FranciscoSandovici, MariaGuo, LilongSantarpino, GiuseppeHakovirta, Harri H.Santovito, DonatoHall, DuaneSbrollini, AgneseHandcock, PhilSchach, ChristianHatoum, HodaSchlüter, Klaus-DieterHawrysz, LilianaSchmucker, JohannesHenderson, DeborahSchoen, FrederickHenein, MichaelSchrickel, Jan WilkoHołda, Mateusz KrystianScott, IanHörer, JürgenSedmera, DavidHorowitz, John D.Sekiguchi, AkikoHoshi, TomoyaSenbonmatsu, TakaakiHossain, Mohammad BakhtiarSergi, Consolato M.Houyel, LucileSerraino, Giuseppe FilibertoHsu, JonghauShah, Nilank C.Hu, DongjianShelton, Elaine L.Huang, HuiShephard, Mary N.Huang, KeSheth, SudipHuang, NganShi, De-LiHubacek, Jaroslav A.Shimoni, SaraHueb, Whady ArmindoShingu, YasushigeHund, ThomasShiono, YasutsuguHwang, Daw-YangSikalidis, AngelosIliou, Marie ChristineSingh, Anand PrakashInoue, KatsujiSirico, FeliceIop, LauraSmith, KellyIshiguchi, HironoriSnyder, Christopher S.Izawa, Kazuhiro P.Soliman, OsamaJackson-Cook, Colleen K.Soma, PrashillaJagielak, DariuszSperling, Silke R.Jang, Sun-JooSpiewak, MateuszJaźwińska, AnnaStanojevic, Dragana M.Jensen, BjarkeStătescu, CristianJiao, KaiStein, Joerg-IngolfJimeno, Cecilia AlegadoStieber, JulianeJordaens, Luc J. L. M.Sturtzel, CaterinaJouk, Pierre SimonSuen, Jacky Y.Joung, BoyoungSugiura, AtsushiJust, SteffenSullivan, Ryan D.Kablak-Ziembicka, AnnaSutanto, HenryKa̧dziela, JacekSzczepańska-Gieracha, JoannaKałka, DariuszSzeiffova Bacova, BarbaraKanda, TatsuoSzekacs, BelaKang, Ju-SeopSzöcs, KatalinKaratzanos, EleftheriosTada, HayatoKasai, TakatoshiTakeda, AtsuhitoKaulitz, RenateTamborini, GloriaKavsak, Peter A.Tappia, ParamjitKeceli, GizemTorella, DanieleKeller, BradleyTribulova, NarcisKelly, RobertTrimarco, BrunoKiliszek, MarekTrindade, FábioKim, Kye HunTuru, GáborKing, Benjamin L.Unterhuber, MatthiasKingsley, MichaelVan Eys, Guillaume J.Kitsara, MariaVanoli, EmilioKlaassen, SabineVaskelyte, Jolanta-JustinaKobayashi, RyotaVendramin, IgorKołodziejczak, MichalinaVenketasubramanian, NarayanaswamyKolodziejski, Pawel AntoniVetrugno, LuigiKoltowska, KaskaVicente, Cristina P.Koshiba-Takeuchi, KazukoVila Sobrino, Xosé AntónKostner, Gert M.Vrachatis, Dimitrios A.Koutouzis, MichaelWachter, RolfKubilius, RaimondasWare, StephanieKumar Mishra, AjayWatanabe, RyuKuroyanagi, HidehitoWaxman, JoshuaKusunose, KenyaWayda, BrianKutikhin, Anton G.Weiss, ChristelKuwata, ShingoWeyand, MichaelLancellotti, PatrizioWięckowski, KrzysztofLansberg, PeterWigle, JeffreyLee, Wei-ChiehWilczek, PiotrLevesque, EricWittlinger, ThomasLi, NingWu, Sean M.Li, SongnanYagishita, AtsuhikoLichtenauer, MichaelYamagishi, HiroyukiLien, Ching-Ling (Ellen)Yamashita, MasashiLin, Yuh-JyhYeh, Jih-KaiLiu, JiandongYutzey, KatherineLombardi, RaffaellaZaffran, StéphaneLonghini, FedericoZanella, Fabian


